# Molecular insights and ADMET profiling of iridoids as candidate leads for erectile dysfunction therapy

**DOI:** 10.1007/s00894-026-06924-z

**Published:** 2026-08-28

**Authors:** Marcos Aurélio Carpina de Oliveira Filho, Jacilene Silva, Victor Moreira de Oliveira, Damião Sampaio de Sousa, Matheus Nunes da Rocha, Márcia Machado Marinho, Emmanuel Silva Marinho

**Affiliations:** 1 Postgraduate Program in Natural Sciences – PPGCN, State University of Ceará, Fortaleza, Brazil; 2 Department of Biological Chemistry, Regional University of Cariri, Crato, Ceará Brazil; 3 Postgraduate Program in Biotechnology, State University of Ceará, Fortaleza, Brazil; 4 Center for Science and Technology/Environmental Studies Nucleus, Ceará State University, Fortaleza, Ceará Brazil; 5 Postgraduate Program in Veterinary Sciences, State University of Ceará, Fortaleza, Ceará Brazil

**Keywords:** Catalposide, Phosphodiesterase, Natural products, PDE-5/PDE-6 selectivity, Erectile dysfunction

## Abstract

**Context:**

Erectile dysfunction (ED) significantly affects male psychosocial health and has an increasing incidence. Although phosphodiesterase 5 (PDEi-5) inhibitors are the first-line therapy, the occurrence of visual side effects resulting from cross-inhibition of the PDE-6 isoform limits their adoption. In this context, the plant *Veronica anagallis-aquatica L.* emerges as a promising source of bioactive therapeutic compounds. This work investigated the inhibitory potential and selectivity of eight iridoid glycosides from this species against PDE-5 and PDE-6. The results identified catalposide as the most potent ligand, exhibiting an affinity energy of -8.6 kcal/mol against PDE-5, surpassing the reference drug Vardenafil (-8.3 kcal/mol) and showing high selectivity towards PDE-6. Trajectory analyses confirmed the robustness of the catalposide-PDE-5 complex, which exhibited smaller structural fluctuations than the commercial control. Despite initial predictions indicating low oral bioavailability, structural optimization through increased sp3 fraction proved to be a viable strategy for enhancing membrane permeability. In conclusion, the findings position catalposide as a promising prototype for the development of more selective and safe drugs for the treatment of ED.

**Methods:**

The two-dimensional structures of the ligands were constructed in Marvin JS and subjected to energy minimization using the semi-empirical PM7 method in MOPAC software. The preparation of macromolecules (addition of polar hydrogens and Gasteiger and Kollman charges) was performed in AutoDockTools. Virtual screening by molecular docking was performed in AutoDock Vina, with exhaustiveness set to 64. The stability of the protein-ligand complexes was evaluated through 500 ns molecular dynamics (MD) simulations using GROMACS 2020.4 software. The systems were solvated with the three-point water model (TIP3P) in a cubic box and described by the CHARMM36 force field. The ligand parameterization was generated via the SwissParam server. The simulations employed the LeapFrog integrator, temperature maintained constant at 310 K via V-rescale thermostat, and pressure coupling maintained at 1 bar using the Parrinello-Rahman method. For predictions of physicochemical properties, pharmacokinetic optimization, and ADMET profile analysis (including hERG toxicity), the predictive servers ADMETlab 3.0, optADMET, ADMET-AI, XenoSite, StopTox, and Pred-hERG were used. Three-dimensional structural complexity was evaluated from the calculation of the MCE-18 function.

## Introduction

Erectile dysfunction (ED) is one of the conditions that most impacts men's health and psychosocial well-being. Characterized by the recurrent inability to achieve or maintain a penile erection sufficient for satisfactory sexual intercourse, its etiology is multifactorial, involving psychological, vascular, hormonal, neurological, and genetic components [1]. Physiologically, an erection depends on a complex neurovascular process that requires adequate blood flow to the penile tissue, hormonal regulation, and stimulation of the nervous system. Factors associated with contemporary lifestyle, such as chronic stress, poor diet, and sedentary behavior, are prevalent causes for the development of ED, the incidence of which also increases with age [2, 3]. In the United States, for example, the prevalence of ED in men aged 75 and older is 52.2%, compared to 17.9% in young people aged 18 to 24 [4]. This results in an annual expenditure, paid directly by Medicare patients, of approximately $696 for emergency treatments using only oral medications (PDEi-5). If we consider more complex treatments such as intraurethral alprostadil, intracavernous injections, and vacuum erection devices (VEDs), for example, the costs already reach thousands of dollars annually [5]. However, there is a recurring increase in cases among younger people, often associated with alcohol and drug abuse and excessive consumption of pornography [6].

The impact of ED transcends the individual, also affecting interpersonal relationships, and is a frequent cause of conflict and separation [7]. In more severe cases, the condition has been associated with suicidal thoughts, highlighting the depth of its psychological impact [8]. Therefore, the search for effective and affordable treatments for ED is an important global public health issue.

The therapeutic approach to ED has undergone a radical transformation with the advent of phosphodiesterase 5 inhibitors (PDE-5 inhibitors). Drugs such as sildenafil (Viagra®), tadalafil, Vardenafil, and avanafil represent first-line oral therapy, relegating other methods, such as alprostadil injections and vacuum devices, to secondary therapies [9, 10]. Phosphodiesterases (PDEs) are a superfamily of 11 classes of enzymes that regulate intracellular levels of the second messengers cyclic adenosine monophosphate (cAMP) and cyclic guanosine monophosphate (cGMP). Inhibition of these enzymes increases cAMP and cGMP concentrations, activating protein kinases A and G, which in turn phosphorylate substrates involved in multiple physiological processes, such as immune response, platelet aggregation, and smooth muscle relaxation [11, 12]. Therefore, PDEi inhibitors are being explored as potential therapeutic agents with diverse activities [13].

Among the isoforms, PDE-5 is selective for cGMP, acting on its hydrolysis, and is predominantly expressed in the smooth muscle of the corpora cavernosa of the penis, where it regulates penile erection. Its inhibition results in the accumulation of cGMP in these tissues, which ultimately promotes muscle relaxation and vasodilation, facilitating the achievement of an adequate erection [14]. This process is fundamental in the treatment of ED, since in many cases penile vascular tone is impaired [15, 16]. However, synthetic PDE-5 inhibitors can exhibit cross-reactivity with structurally similar isoforms, such as PDE-6 (retina), which can lead to side effects such as bluish vision [17, 18]. Therefore, new, more selective PDE-5 inhibitors are crucial for reducing one of the main side effects associated with these medications: bluish vision. With this in mind, natural products emerge as an alternative in the search for new treatments, due to their history of use by various cultures for millennia and their wide variety of compositions.

Historically, natural products have been an invaluable source of bioactive compounds, serving as the basis for the development of numerous drugs [19]. In this context, several plants are traditionally used to treat sexual dysfunctions, such as *Cornus officinalis* [20], *Eucommia ulmoides* [21], and *Morinda officinalis* [22]. They all have in common the fact that they are rich in iridoid compounds, which are characterized by having a general cyclopentopyran form, and are structurally classified according to the presence or absence of intramolecular glycosidic bonds, into iridoid glycosides and non-glycosidic iridoids [23].

The plants known as *Veronica anagallis-aquatica L.*, a European and Asian species whose parts are used in folk medicine for various purposes [24, 25], is also rich in iridoid glycosides [26], a class of compounds with notable biological activities, such as anti-inflammatory, antidiabetic, and anticancer properties [27, 28].

Considering the recurrence of iridoids in plants traditionally used to treat sexual dysfunctions, this study aims to investigate, through molecular modeling techniques, the potential inhibitory action of eight iridoid glycosides, isolated from *Veronica anagallis-aquatica L*., on the PDE-5 enzyme.

Our goal is to evaluate the selectivity of these compounds with respect to PDE-5, in order to select candidates that inhibit this isoform without compromising visual function. By reducing the ability to interact with PDE-6, we seek to avoid interference with cGMP levels in the retina, eliminating the bluish vision caused by the lack of selectivity of current drugs.

## Materials and methods

### Obtaining the structures of proteins and ligands

The RCSB Protein Data Bank (PDB) repository (https://www.rcsb.org/) was used to obtain the structure of PDE-5, which is located under the PDB ID code 1XP0 and is classified as a hydrolase of the organism *Homo sapiens* [29, 30]. PDE-6, on the other hand, can be found with the PDB ID code 5ML6, and is classified as a lipid-binding protein in the organism *Homo sapiens* [31].

**Fig. 1:**
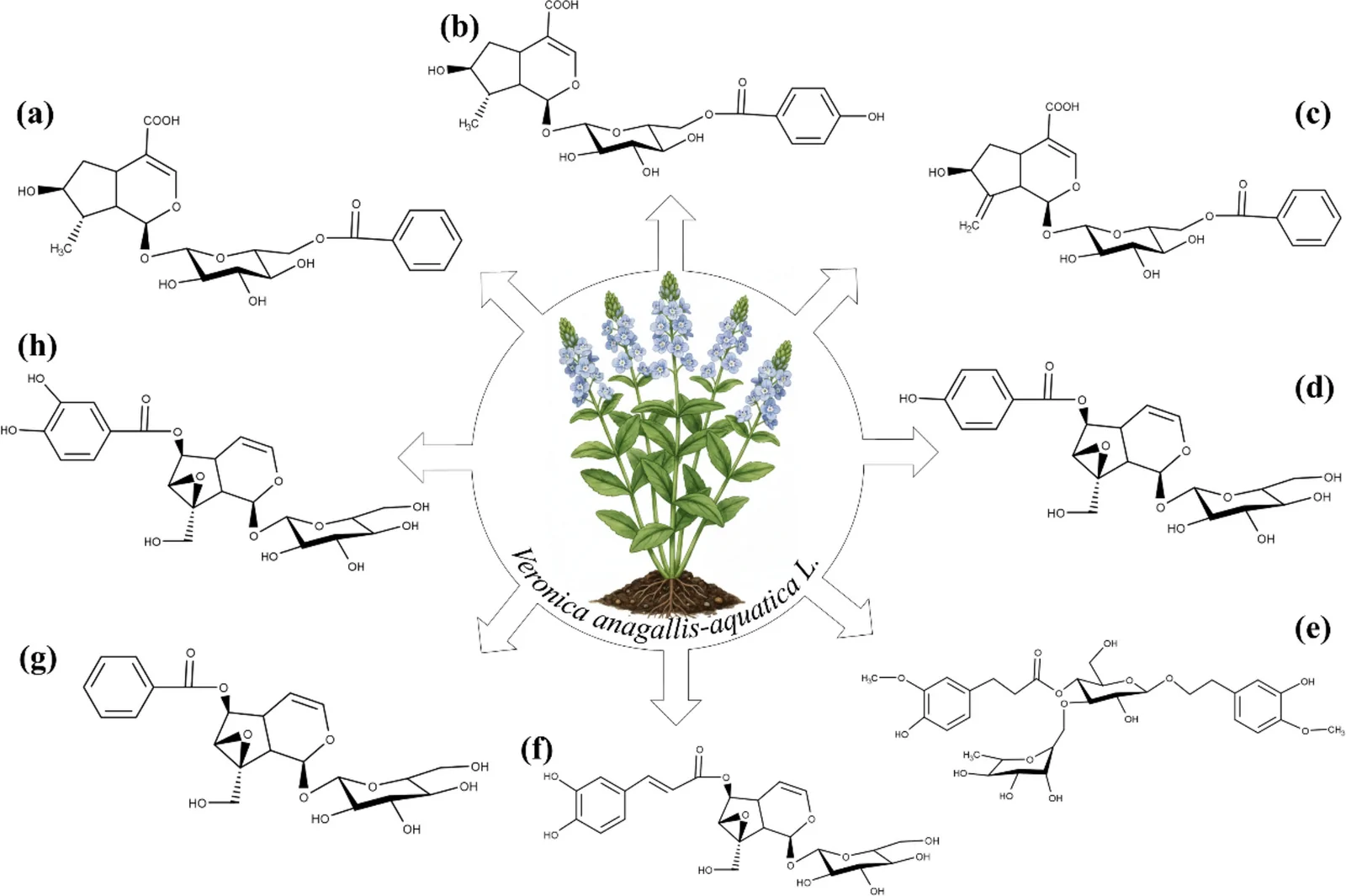
Two-dimensional structure of the iridoids of V. anagallis L., (**a**) aquaticoside A, (**b**) aquaticoside B, (**c**) aquaticoside C, (**d**) catalposide, (**e**) martynoside, (**f**) verminoside, (**g**) veronicoside, and (**h**) verproside [25]

The structures of the ligands were obtained from the work of [25]. The compounds were obtained from ethanolic and aqueous extracts of the aerial parts of the plant *Veronica anagallis-aquatica L.,* collected on the Beytepe campus of Hacettepe University, Ankara, Turkey, in July 2002. The extracts were fractionated, and the compounds were identified by chromatography and spectroscopy methods, allowing the acquisition of the 2D structures of the eight candidate compounds: aquaticoside A, aquaticoside B, aquaticoside C, catalposide, martynoside, verminoside, veronicoside, and verproside (Fig. 1) [25].

### Preparation of ligands and macromolecules for molecular docking

The 2D structures of the compounds were designed using Marvin JS® version 23.11.0 and Chemaxon® software (https://chemaxon.com/marvin). This allowed us to obtain the SMILES (Simplified Molecular Line Entry System) codes for the substances, which were used for the virtual screening step by ADMET. We also obtained the three-dimensional structures of the compounds, which underwent an energy minimization step using MOPAC® software [32], where the most energetically favorable conformation was found using the semi-empirical PM7 [33] method (Parametric Method 7), for all compounds.

The macromolecules were prepared using AutoDockTools® software [34], beginning with the removal of wastewater and other crystallization residues, polar hydrogens and Kollman and Gateiger charges were subsequently added. For the definition of the PDE-5 grid box, where the entire protein was enclosed, a box center was defined at x = −26.124, y = 36.907, and z = 70.546, with dimensions defined at x = 126, y = 126, and z = 126. For PDE-6, the box center was defined at x = −22.888, y = 20.605, z = 8.866, with dimensions defined at x = 40, y = 50, and z = 40. Molecular docking simulations were performed using AutoDockVina® software [35]. For each of the eight ligands, 50 independent docking runs were performed with an exhaustiveness of 64. In each run, the top 20 poses were saved, resulting in a final ensemble of 1000 conformations per ligand. The ProteinsPlus virtual platform (https://proteins.plus/) was used to predict interaction pockets. This platform uses volume, surface area, and depth parameters to predict the regions most likely to be important for the catalytic action of the protein. To analyze the structural quality of the protein model, the PROCHECK tool, SAVES v6.1 (https://saves.mbi.ucla.edu/), was used. This tool provides a Ramachandran plot, which gives us an overview of the structural quality of the model under study.

### Selection of the most promising candidates

The selection of the most promising compounds was conducted as follows: in the first stage, molecular docking of the eight candidate compounds with PDE-5 was performed. This methodology was used to select the compounds with the highest probability of exhibiting a favorable coupling with the target macromolecule. This favorable interaction can be quantified by analyzing the affinity energy (AE) and their respective RMSDs, where the lower the AE, the greater the chance of a favorable interaction. All compounds presented AE and RMSD values ​​within an adequate parameter; therefore, all underwent a second screening process. In this second selection stage, the eight compounds from the first stage were subjected to a screening process using molecular docking, this time with PDE-6, to verify which compounds exhibit the lowest capacity for interaction with this isoform. For this, in addition to high RMSD values and high EA (> −6.0 kcal/mol), the types of interactions with the isoforms were also considered. Each isoform has a set of fundamental residues for its action, some of which are specific to each PDE [29]. Therefore, to ascertain selectivity, we considered that a high number of interactions with specific PDE-5 residues and a reduced number of interactions with the key residues for the functioning of PDE-6 may indicate that the compound has a greater affinity for PDE-5. The low affinity for PDE-6 and poor interaction with the catalytic site may indicate that these compounds have a lower capacity to cause the visual side effects common in PDE-5 inhibitors due to cross-interactions with PDE-6.

Thus, the compounds selected for the following steps, molecular dynamics (MD) and the ADMET study, were those that obtained favorable adverse effects (AEs) with PDE-5, acting as a stimulant in the corpora cavernosa, and that simultaneously possessed a low AE with PDE-6, which may indicate a reduction in vision-related side effects.

### Molecular dynamics (MD)

The stability of the complexes was assessed through MD simulations using the GROMACS 2020.4 software [36]. It was used to perform the simulations with the LeapFrog integrator [37]. The equations of motion were integrated with a time step of 0.001 ps, and no holonomic constraints were applied. The long-range electrostatic interactions were evaluated using the Particle Mesh Ewald (PME) method. Throughout all stages of the 500 ns simulation, temperature and pressure were kept constant for all systems, using the V-rescale method [38]. The Parrinello-Rahman method was used to maintain a constant temperature of 310 K, while the pressure remained constant at 1 bar during the MD simulations [39]. For the solvation step, the three-point water model (TIP3P) was used, adding water molecules to a cubic box with dimensions sufficient to completely enclose the complex [40]. After the addition of water molecules, the system underwent a charge equilibrium step where Na^+^ (182) and Cl^−^ (173) ions were added. The program was configured to use the CHARMM36 force field as the default [41]. The linkers went through a parameterization process via the SwissParam server (https://www.swissparam.ch/).

1 $$RMSD= \sqrt{\frac{1}{n}\sum\limits_{i=1}^{N}{\left({x}_{i1}- {x}_{i2}\right)}^{2} + {\left({y}_{i1}- {y}_{i2}\right)}^{2}+ {\left({z}_{i1}- {z}_{i2}\right)}^{2}}$$

2 $${RMSF}_{atom}= \sqrt{\frac{1}{t}\sum\limits_{i=1}^{t}{\left({x}_{i}^{ref}- {x}_{i}\right)}^{2} + {\left({y}_{i}^{ref}- {y}_{i}\right)}^{2}+ {\left({Z}_{i}^{ref}- {Z}_{i}\right)}^{2}}$$

MD simulations provide crucial information about the stability of L-P interactions and are fundamental for studying the physicochemical properties and dynamic behavior of chemical systems [42], being a fundamental step in the validation of computational studies. For the evaluation of system stability, their RMSDs (Mean Squared Deviation) are used [43] (Eq. 1). These parameters were calculated, providing insight into the structural stability of the studied systems. They measure the average structural difference between the structures, where low values indicate high structural similarity and low structural deformation, while high RMSD values indicate a high average variation between the structures. In other words, there was a greater degree of structural deformation in the system at the end of the 500 ns MD simulation [44].

An inherent limitation of static molecular docking is the treatment of receptors as rigid structures, which neglects the conformational flexibility inherent in biological macromolecules. To mitigate this limitation and provide greater robustness to the study, Molecular Dynamics (MD) was used to calculate Mean Squared Fluctuations (RMSF). This descriptor quantifies the local atomic displacement relative to a reference structure along a 500 ns path, allowing the evaluation of the thermodynamic stability and plasticity of amino acid residues after complexation with the ligand, as described by Eq. 2 [45, 46], providing regions that undergo deformation or are stabilized due to the presence of the ligand, thus indicating regions responsible for the enzyme's catalytic action.

To estimate the binding affinity of protein-ligand complexes, we employed the interaction potential energy (IPE) formalism correlated with the linear interaction energy (LIE) method. This approach is based on the statistical sampling of non-bonded potential energies obtained from molecular dynamics trajectories, evaluating the ligand in two distinct physical states: free in aqueous solution and complexed within the macromolecule's active site [47]. The change in binding free energy (∆G_bind_) is calculated using Eq. 3:

3 $$\Delta \mathrm{G}_{\mathrm{b}\mathrm{i}\mathrm{n}\mathrm{d}} = \upalpha (\langle {V}_{vdw}\rangle bound - \langle {V}_{vdw}\rangle free) +\upbeta (\langle {V}_{alec}\rangle bound -\langle {V}_{elec}\rangle free) + \upgamma$$

Here, Vvdw corresponds to the potential energy associated with van der Waals interactions between the ligand and its environment; Velec is the electrostatic interaction potential component (Coulomb potential); α and β are empirical coefficients for each scale used to weight the van der Waals and electrostatic contributions, respectively; and γ is an empirical fitting constant associated primarily with hydrophobic cavity effects and active-site desolvation.

### ADMET prediction

#### Physicochemical properties and molecular complexity

For the analysis of structural complexity and synthetic accessibility, the number of aromatic (AR) and non-aromatic (NAR) rings, chiral and spirocyclic centers, as well as the sp3 distribution between cyclic (Cyc) and acyclic (Acyc) structures, were combined in the function of "Medicinal Chemistry Evolution", 2018 (MCE-18):

4 $$MCE18=(AR+NAR+Chiral+Spiro+\left({Fsp}^{3}+Cyc-Acyc/1+ {Fsp}^{3}\right){Q}^{1})$$

The resulting classification is presented in the following order:

- Scores below 45: compounds with low 3D structural complexity;
- Scores between 45 and 63: structures that basically follow the trends currently observed in medicinal chemistry;
- Scores between 63 and 78: high structural similarity to compounds registered in patents;
- Scores above 78: high degree of structural novelty and 3D complexity [48].

The filters in question are available in the Medicinal Chemistry predictor of the ADMETTlab 3.0 server (https://ADMETtlab3.scbdd.com/). The results were correlated with lipophilicity descriptors, expressed as octanol-water partition coefficient (logP) and distribution coefficient at physiological pH, estimated using the optADMETT predictor (https://cadd.nscc-tj.cn/deploy/optADMETt/).

#### Pharmacokinetic optimization

The optADMETT service (https://cadd.nscc-tj.cn/deploy/optADMETt/) was configured to optimize the physicochemical attributes of logP and logD based on the substitution of polar groups with Fsp3-rich alkyl moieties, aiming to optimize absorption, distribution, metabolism, and excretion (ADMET) descriptors in vitro, which include apparent cell permeability rate (Papp) in colorectal adenocarcinoma cells (Caco-2), oral bioavailability, volume of distribution of steady state (VDss), and intrinsic hepatic clearance rate (CLint,u). The results were related to the ADMET profile estimated by the AI-guided data similarity test in relation to drugs deposited in the DrugBank database, using the ADMETT-AI predictor (https://ADMETt.ai.greenstonebio.com/).

#### hERG safety and metabolic stability

The prediction of the metabolism site was performed using the XenoSite (https://xenosite.org/) and StopTox (https://stoptox.mml.unc.edu/) servers. The prediction of cardiotoxicity by inhibition of human Ether-a-go-go-Related Gene (hERG) channels was performed based on the Pred-hERG predictor (https://predherg.labmol.com.br/). The responses in two-dimensional graphic signatures, expressed in probability heatmaps, were related to the inhibitory concentration (IC50) of hERG data and metabolic stability descriptors, expressed in liver microsome clearance (CLMicro) and hepatocyte clearance (CLHepa) [48].

## Results and discussion

### Validation of protein structure

**Fig. 2:**
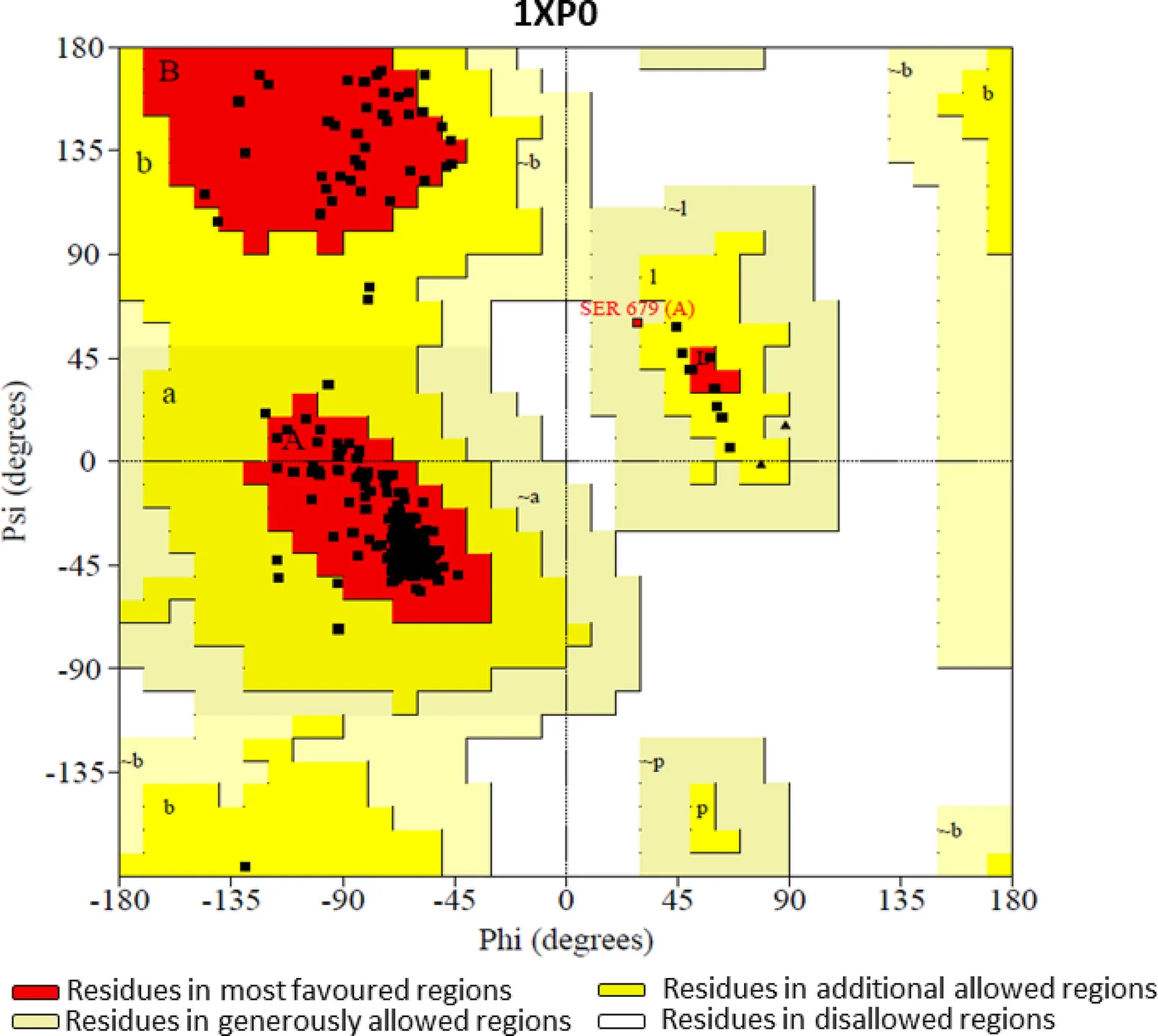
Ramachandran plot of phosphodiesterase 5 (PDE-5), residues in most favored regions [A, B, L] 93.5%, residues in additional allowed regions [a, b, l, p] 6.2%, residues in generously allowed regions [~ a, ~ b, ~ l, ~ p] 0.3%, residues in disallowed regions 0.0%

To assess the structural quality of the protein (PDE-5), its Ramachandran plot was delineated using the PROCHECK tool, SAVES v6.1 (https://saves.mbi.ucla.edu/), allowing for the analysis of the dihedral angles of the peptide bonds of each residue in the structure. The analysis of the torsion angles, ɸ (phi) and Ψ (psi), indicates which regions of the protein have steric hindrance. Therefore, the Ramachandran plot is a way to evaluate the quality of the protein model, with a percentage above 90% of residues in favorable regions demonstrating higher structural quality [49, 50]. The Ramachandran plot of PDE-5 (Fig. 2) shows that 93.5% of the residues are located in favorable regions of the protein, i.e., regions where there are no steric hindrances. We can also observe that no residues are present in unfavorable regions of the protein.

Therefore, we can conclude that the PDE-5 model is structurally reliable, making it a suitable model for computational studies.

### Prediction of cavities

**Fig. 3:**
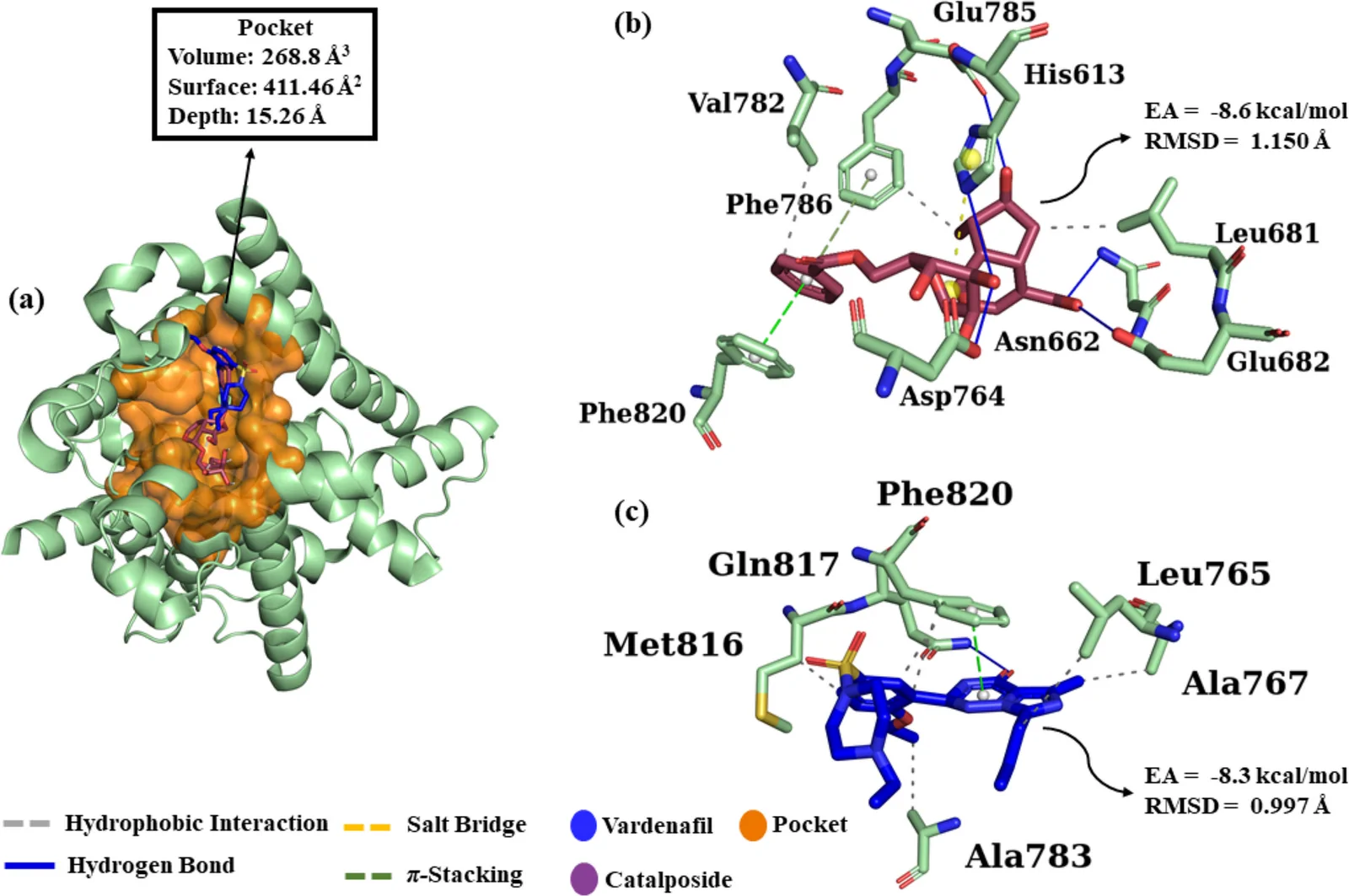
Mapping of the active site and molecular interactions in PDE-5. (**a**) Visualization of the catalytic pocket showing the volume and depth of the cavity. (**b**) Interaction profile of Catalposide with key residues of the active site. (**c**) Binding mode of the reference drug, Vardenafil, at the catalytic site of PDE-5

The ProteinsPlus platform provided the protein region most likely to be important for its catalytic action, this pocket being the region of greatest interest for our studies, as interactions in this area of ​​the protein may indicate that the compound under study has some action on the target. A cross-analysis with data from the literature [29], showed an excellent correlation with the platform data, where we can verify that practically all the residues from the pocket provided by the platform (Tyr612, His613, His617, His653, Asp654, His657, Ser661, Asn662, Leu681, Glu682, Thr723, Asp724, Leu725, Asp764, Leu765, Ser766, Ala767, Ile768, Gln775, Ile778, Ala779, Val782, Phe786, Gln817 and Phe820), in orange in Fig. 3a, are part of the PDE-5 active site reported in the literature [29]. This result demonstrates the validity of the data provided by the platform.

### Molecular docking studies with PDE-5 and PDE-6

Molecular docking, using PDE-6 as the main selection filter, showed that the compound that interacted least with this isoform was catalposide, with an average EA of −6.48 kcal/mol, 46.7% of the poses presented EA and RMSD outside the standards adopted as acceptable for molecular docking studies (AE ≤ −6.0 kcal/mol and RMSD < 2.0 Å). We also observed a reduced number of interactions with residues such as Glu88, Trp32, Trp90, Tyr149, Cys56, Arg61, Gln78, Met117, and Met118, which make up the delta subunit of PDE-6 and are fundamental to the action of this isoform. This indicates that this compound is the candidate with the lowest potential for interaction with the protein, suggesting a lower capacity to cause vision-related side effects.

Analysis of molecular docking interactions with PDE-5 showed that iridoids exhibited a large number of interactions (Table 1) with the active site of PDE-5 [29] (His613, His617, His653, Asp654, His657, Asn662, Met681, Glu682, Asp764, Met816, Gln817, Phe820, Gly659, Met805, Tyr612, Leu765, Ile778, Ala779, Val782, Ala783, Leu804, Ile813, Asn661, Glu785, Thr802, Leu725, Gln775, Gln789, Asp724, Ile768 and Phe786), the predominant interactions were hydrophobic, H-Bond, π-stacking and salt Bridges. We can observe that catalposide showed two hydrophobic interactions with Phe786 (3.60 Å and 3.43 Å) (Fig. 3b), this being an important residue of the active site, this residue interacts in the same way with Vardenafil [29] (Fig. 3c), indicating the relevance of this residue to the action of PDE-5, this being a specific residue for this isoform. We also observed five H-bond interactions with the residues Asn662 (2.76 Å and 2.00 Å), Asp724 (2.06 Å), and Leu725 (3.03 Å) (Fig. 3b). These residues are located in the active site of PDE-5 and are specific to this isoform. This interaction may indicate a greater specificity of the catalposide towards PDE-5. Hydrogen bond interaction was observed with Thr723 (2.62 Å), two π-stacking interactions were also observed, with the residues Phe786 (5.38 Å) and Phe820 (388 Å) (Fig. 3b), both of which are part of the active site of PDE-5. We highlight that these residues are also specific to this isoform. Three salt-type interactions were also observed, bridges with the residues His613 (5.35 Å and 5.33 Å) and His657 (5.46 Å) (Fig. 3b), both belonging to the active site of PDE5, but unlike the other interactions, these three are formed by residues that are conserved among all PDEs.

**Table 1:** RMSD and types of interactions formed between ligands and the PDE-5 in the molecular docking simulations

Ligand	RMSD (Å)	Interaction Type	Residue (Distance in Å)
Aquaticoside A	1.607	Hydrophobic	Leu681(3.58), Val782(3.93),Phe786(3.25)
		H-Bond	His613(3.24), Asn662(1.83), Glu682(2.71), Asp764(1.95), Glu785(2.68), Glu785(2.58)
		π-Stacking	Phe786(5.41), Phe820(3.74)
		Salt Bridges	His613(5.03)
Aquaticoside B	1.495	Hydrophobic	Leu681(3.62), Val782(3.77), Phe786(3.29), Phe820(3.63), Phe820(3.71)
		H-Bond	His613(3.22), Asp654(2.84), Asn662(3.04), Asn662(1.88), Asn662(3.02), Thr723(2.77), Asp764(2.16), Gln775(1.72),Gln775(3.49), Glu785(2.78)
		Salt Bridges	His613(5.13)
Aquaticoside C	1.868	Hydrophobic	Leu725(3.80), Ala767(3.87), Ile768(3.71), Val782(3.78), Phe820(3.73)
		H-Bond	613His(3.18), Thr723(2.54), Leu725(2.88), Ala726(2.25), Asp764(2.45)
		Salt Bridges	613His(4.92), His613(4.07)
Catalposide	1.150	Hydrophobic	Phe786(3.60), Phe786(3.43)
		H-Bond	Asn662(2.76), Asn662(2.00), Thr723(2.62), Asp724(2.06), Leu725(3.03)
		π-Stacking	Phe786(5.38), Phe820(3.88)
		Salt Bridges	His613(5.35), His613(5.33), His657(5.46)
Martynoside	1.495	Hydrophobic	Asn662(3.95), Gln663(3.79), Leu725(3.46), Leu765(3.75), Val782(3.87), Val782(3.69), Phe786(3.94), Phe820(3.72), Phe820(3.89)
		H-Bond	His657(2.97), Asn662(2.69), Glu682(2.11)
		Salt Bridges	His613(4.72)
Verminoside	1.582	Hydrophobic	Val782(3.63), Phe786(3.54), Phe820(3.96), Phe820(3.88)
		H-Bond	His613(2.86), His657(2.14), Asn662(2.17), Glu682(1.93)
		Salt Bridges	His613(4.85), His613(4.42)
Veronicoside	1.406	Hydrophobic	Tyr612(3.79), Val782(3.69), Val782(3.62), Phe786(3.95), Phe786(3.17), Phe820(3.89), Phe820(3.44)
		H-Bond	His657(2.76), Asn662(2.75), Asn662(2.07), Glu682(1.76), Thr723(2.26), Leu725(3.13)
		Salt Bridges	His613(5.33), His613(4.10)
Verproside	1.083	Hydrophobic	Phe786(3.59), Phe786(3.42)
		H-Bond	Asn662(2.80), Asn662(2.04), Glu682(2.98), Leu725(3.00), Asp764(2.45), Met816(3.07), Gln817(3.30), Gln817(2.43)
		π-Stacking	Phe786(5.39), Phe820(3.88)
		Salt Bridges	His613(5.32), His613(5.30), His657(5.49)

The catalposide presented EA parameters considered satisfactory for molecular docking studies, with an EA of −8.6 kcal/mol and an RMSD of 1.150 Å (Fig. 3b). These values ​​are similar to those of the cocrystallized ligand, Vardenafil, which has an EA of −8.3 kcal/mol and an RMSD of 0.997 Å (Fig. 3c). These values ​​demonstrate that the affinity of catalposide for PDE-5 is close to the affinity of the vardenafil-PDE-5 complex. These data indicate that catalposide has high potential as a PDE-5 inhibitor. This is demonstrated by the large number of interactions at the enzyme's active site, with these interactions occurring in the same region as those performed by vardenafil. Furthermore, the selectivity of the catalposide-PDE-5 complex was close to that of the complex formed with the cocrystallized drug. This can be verified by the equivalent active interactions between the two complexes. In addition, catalposide showed several favorable interactions with residues that are exclusive to PDE-5, indicating low cross-interaction with other isoforms of the PDE family and suggesting a lower incidence of side effects. Thus, the molecular docking study demonstrated the great potential of catalposide against PDE-5, while also showing low affinity for PDE-6, which is indicative of its low tendency to cause vision-related side effects.

Considering that molecular docking provides only a static snapshot of the interaction, the study proceeded to Molecular Dynamics (MD) simulations. This step aimed to evaluate the thermodynamic stability and integrity of the interaction networks established in the Catalposide–PDE-5 and Vardenafil–PDE-5 complexes over time. Additionally, analysis of the protein in its free state (apoenzyme) was performed to serve as a structural control, allowing the identification of conformational changes induced by the presence of ligands in an explicit solvent environment.

### Molecular dynamics evaluation: stability, interactions, and thermodynamics

To rigorously validate the molecular docking predictions and evaluate the selectivity profile of the compounds, 500 ns Molecular Dynamics (MD) simulations were performed. The analysis focused on structural stability (RMSD and RMSF), specific intermolecular contacts (hydrogen bond occupancy), and was definitively supported by thermodynamic calculations using the Interaction Potential Energy (IPE) method.

#### RMSD, RMSF, and structural stability: PDE-5 vs. PDE-6

**Fig. 4:**
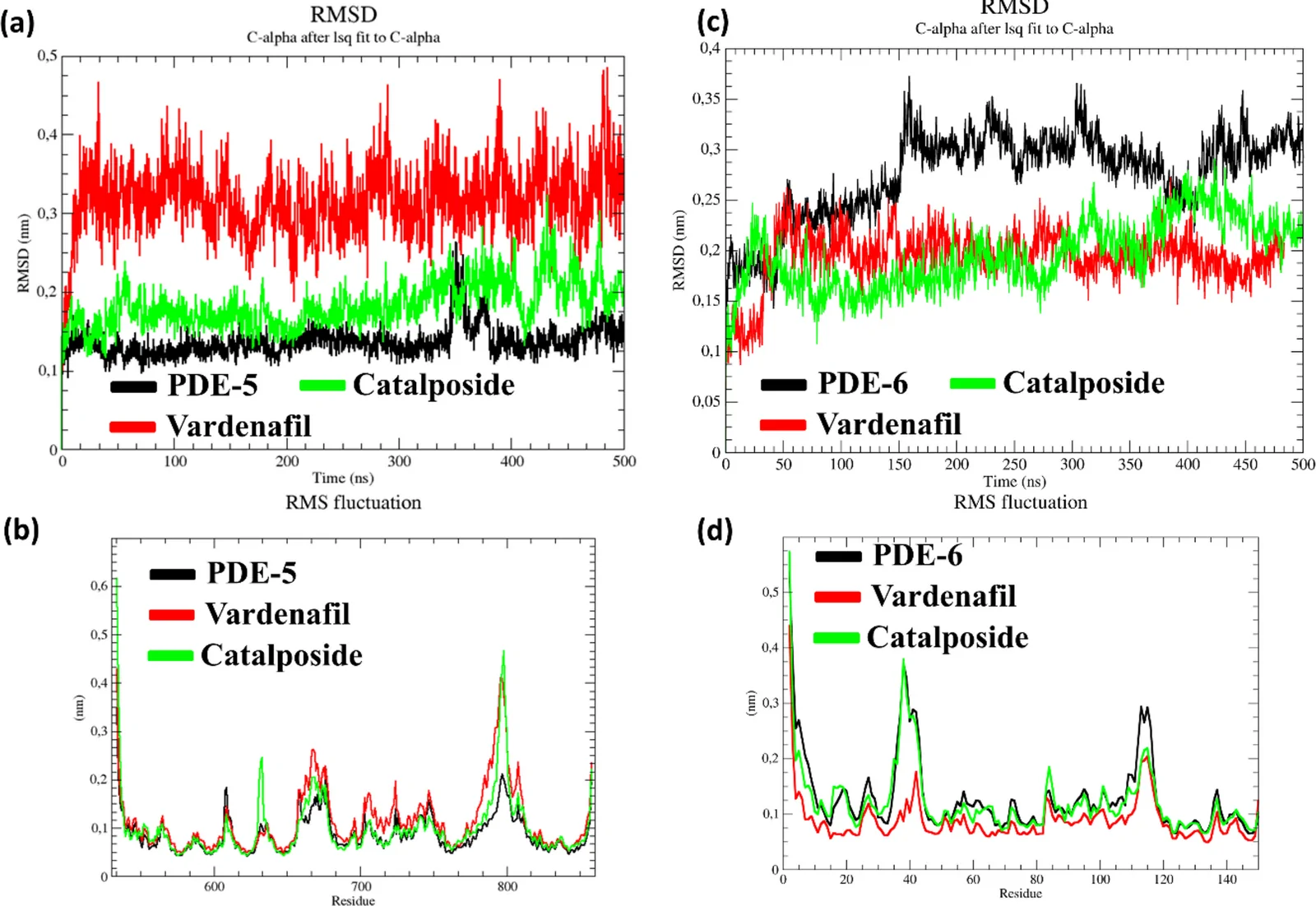
Monitoring of molecular dynamics trajectories over 500 ns to evaluate the structural stability of PDE-5 and PDE-6 isoforms and their respective complexes. (**a**) Evolution of the Root-Mean-Square Deviation (RMSD) and (**b**) Root-Mean-Square Fluctuation (RMSF) profile of amino acid residues for the therapeutic target PDE-5. (**c**) Evolution of RMSD and (**d**) RMSF profile demonstrating high flexibility for the off-target isoform PDE-6. For all plots, the lines represent: the apoenzyme in its free state (black), the complex with the reference drug Vardenafil (red), and the complex with the natural iridoid Catalposide (green)

The Root Mean Square Deviation (RMSD) was calculated to assess the global thermodynamic stability of the complexes throughout the 500 ns simulation. Regarding the therapeutic target (PDE-5), the structure of the free apoenzyme (Fig. 4a, in black) remained virtually unchanged, showing only a single variation peak in the 337–357 ns range. When complexed with catalposide (Fig. 4a, in green), the enzyme proved remarkably stable throughout the period, exhibiting only minor variations in the 412–432, 434–454, and 466–486 ns intervals. In comparison, the complex with Vardenafil (Fig. 4a, in red) also proved stable but showed a slightly higher RMSD value and a significant initial variation of approximately 0.25 nm before stabilizing. This indicates that, overall, the iridoid possesses a stabilizing capacity comparable or even superior to that seen with the primary target.

The critical distinction explaining the selectivity emerges from the analysis of the off-target isoform (PDE-6). The isolated PDE-6 apoenzyme (Fig. 4c, in black) exhibited high structural instability, with a significant jump at approximately 150 ns (reaching about 0.37 nm) and sustained high fluctuations ranging between 0.25 and 0.35 nm until the end of the trajectory. When Vardenafil binds to PDE-6 (Fig. 4c, in red), the RMSD drops significantly, stabilizing in the lower range of 0.15 to 0.20 nm particularly between 300 and 500 ns demonstrating that the reference drug binds firmly and stabilizes this isoform, thereby explaining its cross-reactivity. In stark contrast, the Catalposide-PDE-6 complex (Fig. 4c, in green) exhibited much larger RMSD fluctuations than Vardenafil, closely mimicking the unstable nature of the free enzyme, with prominent peaks in the 10–30 ns, 320–330 ns, and 380–420 ns intervals reaching approximately 0.27 nm.

While RMSD assessed global stability, Root Mean Square Fluctuation (RMSF) was used to quantify the plasticity and local displacement of specific amino acid residues. In the therapeutic target (PDE-5, Fig. 4b, in black), the analysis revealed that the residue range 673–688 maintained high variations across all systems, suggesting an area of intrinsic enzyme flexibility independent of the ligand. Notably, both the complex with catalposide (Fig. 4b, in green) and the one with vardenafil (Fig. 4b, in red) exhibited very similar fluctuation peaks in domains crucial for catalytic activity, specifically in the ranges 783–795, 673–686, 656–668, and 809–821. Critical residues such as His657, Asn662, Met681, and Glu682 are found in these regions. The fact that catalposide mimics vardenafil's vibrational profile at residues determining anchoragesuch as Val782, Ala783, Ile813, Glu785, and Phe786 supports its inhibitory efficacy at the active site. Furthermore, the overall average fluctuation was lower in the catalposide complex, reinforcing its superior conformational stability within PDE-5.

Conversely, in the off-target isoform (PDE-6), the RMSF plot clearly demonstrated the natural candidate's lack of affinity. Free PDE-6 (Fig. 4d, black) exhibited regions of very high fluctuation, notably residues 35–45 (peak of approximately 0.29 nm) and 110–120 (peak of approximately 0.29 nm). Vardenafil (Fig. 4d, red) strongly suppressed these local fluctuations, reducing the peaks to approximately 0.18 nm and 0.20 nm, respectively. Catalposide completely failed to restrict PDE-6 flexibility (Fig. 4d, green), tracing almost the exact same fluctuation profile as the free enzyme and even exceeding the apoenzyme's flexibility in the 35–45 region by reaching a high peak of about 0.38 nm. This inability to stabilize intrinsically flexible domains is a strong thermodynamic indicator of its weak affinity for PDE-6.

#### Analysis of hydrogen bond occupancy in systems with PDE-5

**Fig. 5:**
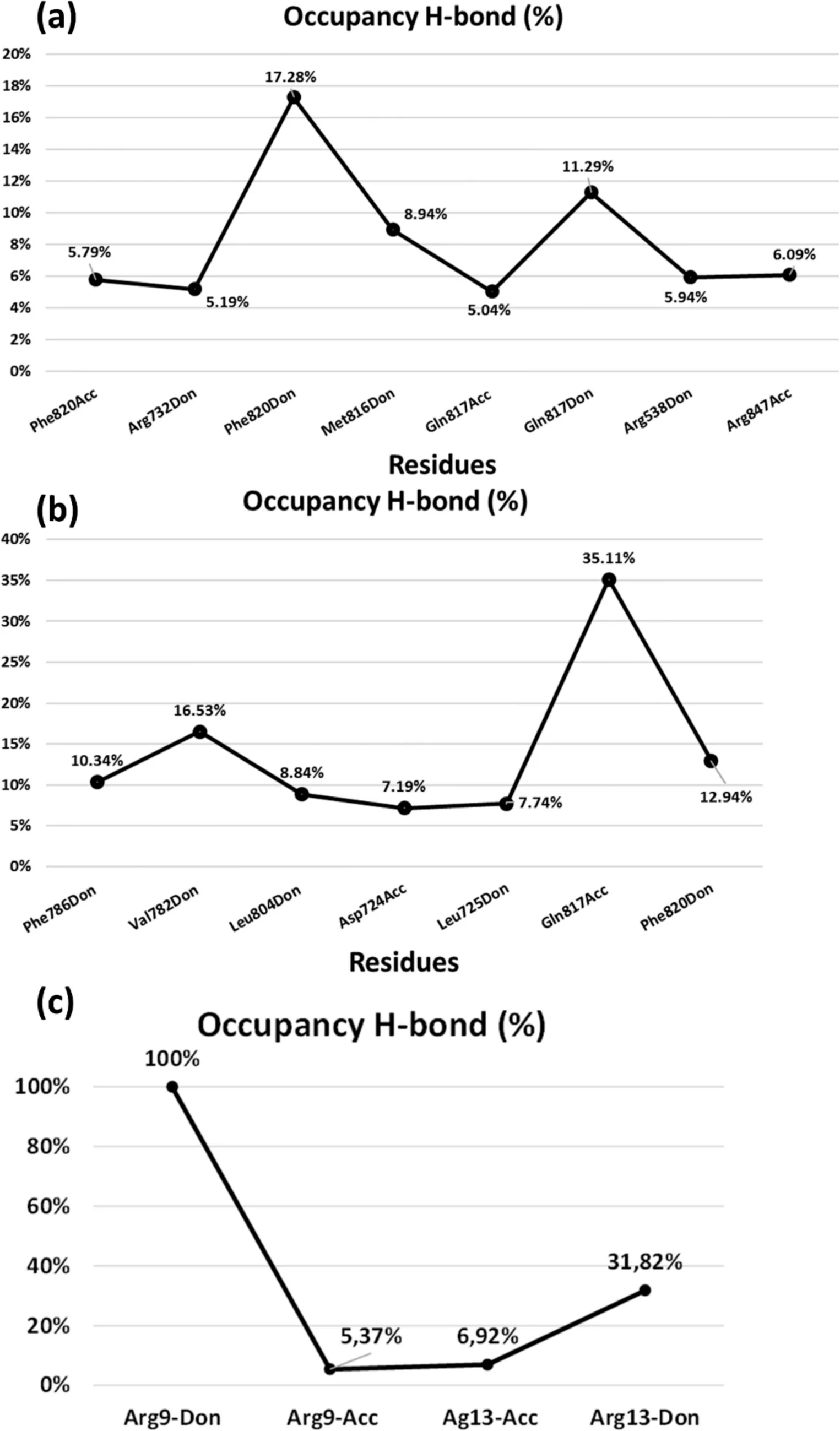
Percentage results for hydrogen bond occupancy (occurrence frequency ≥ 5%) formed between ligands and amino acid residues throughout the 500 ns simulation period. (**a**) Interaction network of the Vardenafil-PDE-5 complex; (**b**) Interaction network of the Catalposide-PDE-5 complex; and (**c**) Cross-reactivity interactions in the Vardenafil-PDE-6 complex. (Explanatory note: The Catalposide-PDE-6 complex was not plotted because it exhibited only a single significant interaction throughout the trajectory an acceptor bond with residue Glu88, with an occupancy of only 19.78%)

To determine the strength of the interaction between the PDE-5-catalposide and PDE-5-vardenafil complexes, the degree of hydrogen bond occupancy between the protein and the respective ligands was analyzed (Fig. 5). For this analysis, only occupancies greater than or equal to 5% were considered.

Hydrogen Bond Occupancy Analysis To determine the strength and persistence of the interactions underlying the RMSD and RMSF behaviors, hydrogen bond occupancy was analyzed, considering values ​​greater than or equal to 5%. Regarding interactions with the PDE-5 active site (Fig. 5a, b), residue Phe820 (acting as a hydrogen donor), essential for forming hydrophobic interactions to anchor inhibitors showed the highest occupancy in the Vardenafil complex (17.28%, Fig. 5a) and also exhibited a strong interaction with catalposide (12.94%, Fig. 5b), confirming that the iridoid reaches the same hydrophobic cavity as the reference drug. A substantial difference favoring the natural product was observed with residue Gln817, which is crucial for selective action on the enzyme. In the catalposide complex, Gln817 (acting as an acceptor) achieved a massive occupancy of 35.11%, compared to only 5.04% for Vardenafil. Additionally, catalposide formed a persistent network of interactions with Asp724 (7.19%), Val782 (16.53%), Leu725 (7.74%), Leu804 (8.84%), and Phe786 (10.34%), corroborating its high capacity to bind firmly to the catalytic site.

The selectivity hypothesis is definitively confirmed by the binding data regarding PDE-6 (Fig. 5c). Vardenafil (Fig. 5c) maintained robust, high-occupancy interactions with this off-target, reaching 100% occupancy with Arg9 (donor) and 31.82% with Arg13 (donor). In contrast, catalposide formed only a single notable hydrogen bond with PDE-6 throughout the entire 500 ns simulation: an isolated interaction with Glu88 (acceptor), achieving an occupancy of just 19.78%. The near-total absence of a stable network of intermolecular hydrogen bonds with PDE-6 consistently explains the overall instability observed in the RMSD and the extremely high residual flexibility demonstrated by the RMSF, indicating that catalposide acts as a highly selective inhibitor with significantly reduced risks of causing the visual side effects associated with vardenafil.

#### Thermodynamic analysis: interaction potential energy (IPE)

To quantify and consolidate theoretical observations regarding structural stability and the nature of intermolecular interactions, the binding free energy change (ΔG_bind) of the complexes was calculated using the Interaction Potential Energy (IPE) method (Table 2) based on simulation trajectories. The thermodynamic results provided strong indications regarding the affinity and selectivity profile of the iridoid.

**Table 2:** Interaction potential energy

Term	∆G_bind_ (kj/mol)
PDE-6-Vardenafi	+ 21.3538
PDE-6-Catalposide	+ 4.67128
PDE-5-Vardenafi	+ 68.2002
PDE-5-Catalposide	−27.0575

Regarding the primary therapeutic target, the PDE-5–catalposide complex stood out by exhibiting a significantly negative predicted binding free energy (ΔG_bind = −27.0575 kJ/mol). This value suggests that the interaction between the natural product and the PDE-5 active site tends to be a thermodynamically spontaneous and favorable process, corroborating the overall stability indicated by the RMSD and the hydrogen-bond-guided binding. In contrast, the PDE-5–vardenafil complex yielded a positive ΔG_bind (+ 68.2002 kJ/mol), indicating that, under the conditions evaluated in the in silico model, the accommodation of the reference drug in its stabilized pose may entail a considerably higher energy cost.

Strong indications regarding the safety profile emerged from the theoretical thermodynamic evaluation against the off-target isoform (PDE-6). With PDE-6, both ligands exhibited positive binding free energies, suggesting non-spontaneous processes and unfavorable affinities. The PDE-6–catalposide complex showed a ΔG_bind of + 4.67128 kJ/mol, whereas the PDE-6–vardenafil complex recorded + 21.3538 kJ/mol.

The thermodynamic discrepancy predicted for catalposide suggesting high spontaneity and affinity for PDE-5 (−27.0575 kJ/mol), in direct contrast to its likely unfavorable interaction with PDE-6 (+ 4.67128 kJ/mol) provides a quantitative basis pointing to a promising selectivity profile. These free-energy calculations align consistently with the root-mean-square fluctuation (RMSF) data and low hydrogen-bond occupancy, thermodynamically indicating catalposide's inability to stabilize the visual isoform. Collectively, these computational data reinforce the iridoid's potential as a prototype inhibitor with a safer profile regarding typical side effects (such as cyanopsia), thereby justifying further experimental investigation.

### Multiparametric ADMET analysis

#### Physicochemical properties and molecular complexity

**Fig. 6:**
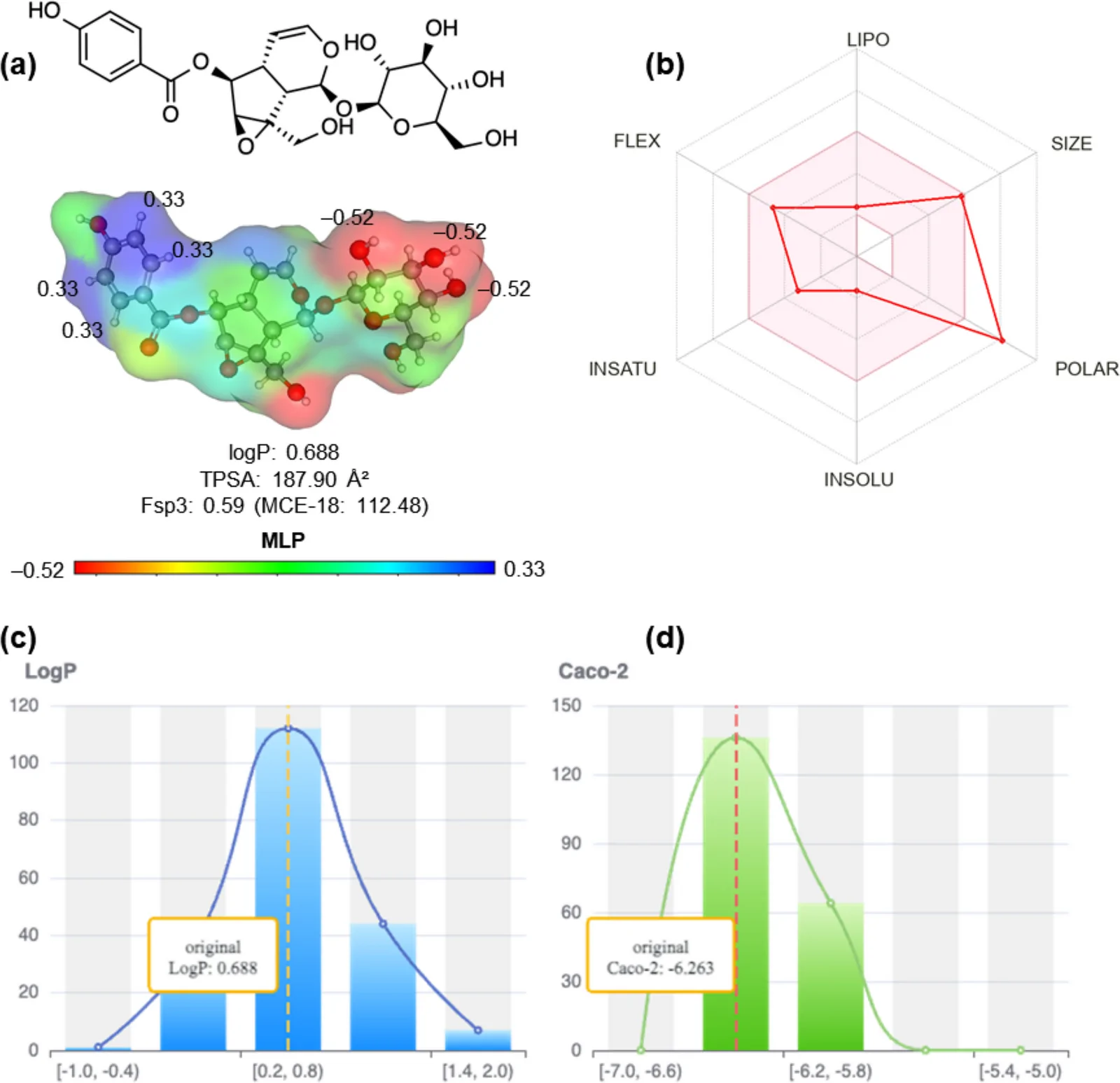
Physicochemical profile and ADMET of the compound. (**a**) Chemical structure and molecular lipophilic potential (MLP) map, highlighting hydrophobic (blue) and hydrophilic (red) regions. Values of logP (0.688), TPSA (187.90 Å2), and sp3 carbon fraction (Fsp3: 0.59) are indicated. (**b**) Radar plot illustrating the suitability of the compound to the oral bioavailability chemical space (pink area). The axes represent lipophilicity (LIPO), size (SIZE), polarity (POLAR), insolubility (INSOLU), unsaturation (INSATU), and flexibility (FLEX). (**c**) Probability distribution of logP compared to the reference dataset. (**d**) Prediction of permeability in Caco-2 cells (logPapp = −6.263 cm/s), indicating the intestinal absorption potential of the compound

In the molecular lipophilicity potential (MLP) map, it is possible to observe that the glycoside structure of Catalposide constitutes a more water-soluble molecular surface (red color spectra), which strongly contributes to the formation of a polar surface (TPSA) estimated at 187.90 Å^2^ (Fig. 6a) [51], while the aromatic region constitutes a more hydrophobic molecular surface (blue color spectra), resulting in a logP on the order of 0.688. In the oral bioavailability radar, it is possible to note that the compound is more polar than considered ideal to ensure good human intestinal absorption (HIA), which decreases as the TPSA increases from 140 Å^2^ (Fig. 6b) [52, 53]. The data similarity test showed that Catalposide exhibited similarity with at least 100 compounds from the CBDD Group database that have logP values ​​in the range of 0.2 and 0.8 (Fig. 6c). Furthermore, the compound showed a predicted logPapp Caco-2 value on the order of −6.263 cm/s, which is good but below ideal for ensuring efficient passive diffusion in the gastrointestinal tract, showing data similarity with at least 120 compounds from the database (Fig. 6d) [54].

#### Pharmacokinetics optimization

Analysis of the physicochemical properties revealed that the compound Catalposide has a calculated Fsp^3^ of 0.59, indicating a 3D structural complexity of an essentially saturated compound, resulting in an MCE-18 score of approximately 112.48, indicating a considerable degree of structural novelty (Fig. 6a). The Fsp^3^ parameter is the target of structural modifications in new ADMET predictive models because they induce the optimization of pharmacokinetic attributes associated with solubility, permeability, and oral absorption [55].

**Fig. 7:**
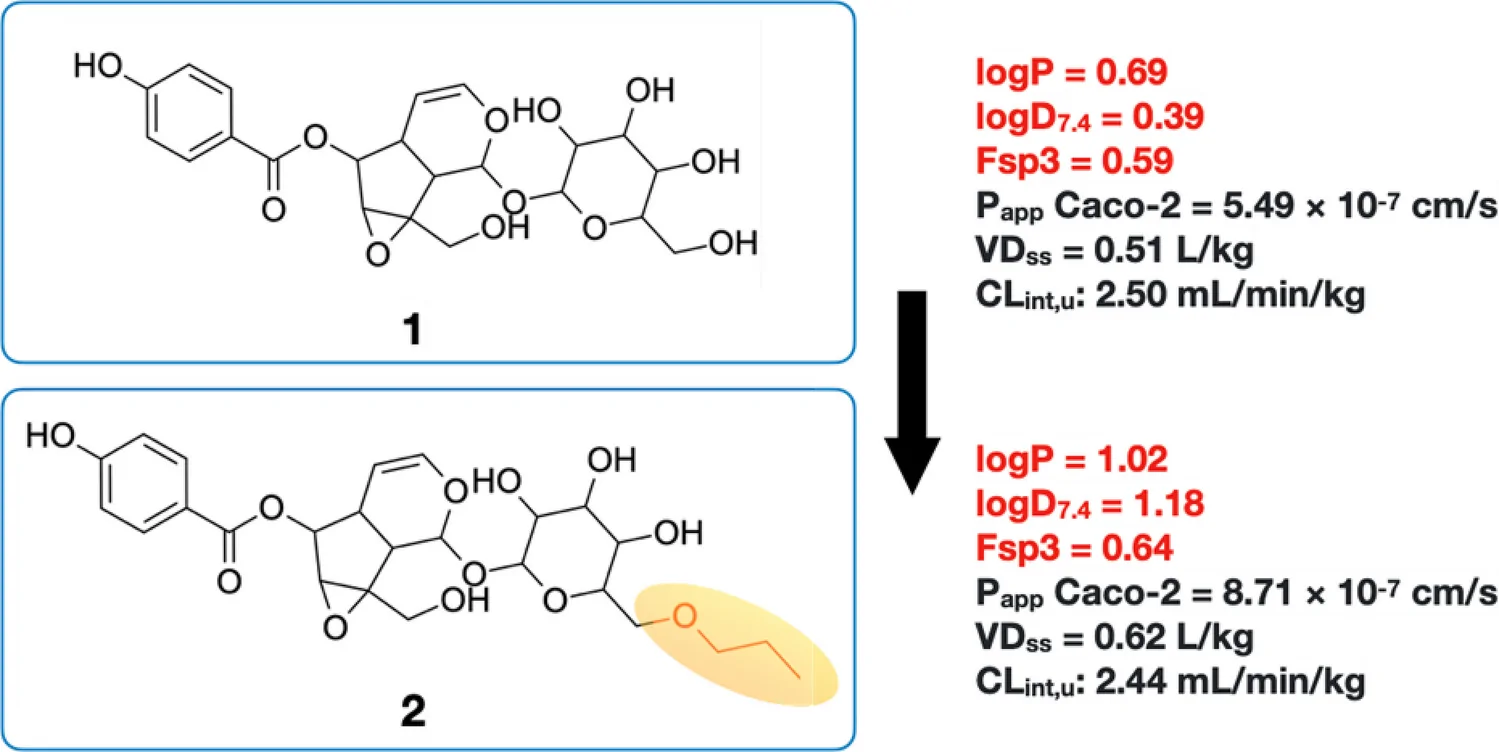
Structural optimization strategy and impact on ADMET properties. Differences in logP, logD, Fsp3 values, Caco-2 permeability, volume of distribution, and clearance between prototype 1 and analog 2. The orange highlight indicates the location of the molecular alteration that favored the increase in lipophilicity and apparent permeability

**Fig. 8:**
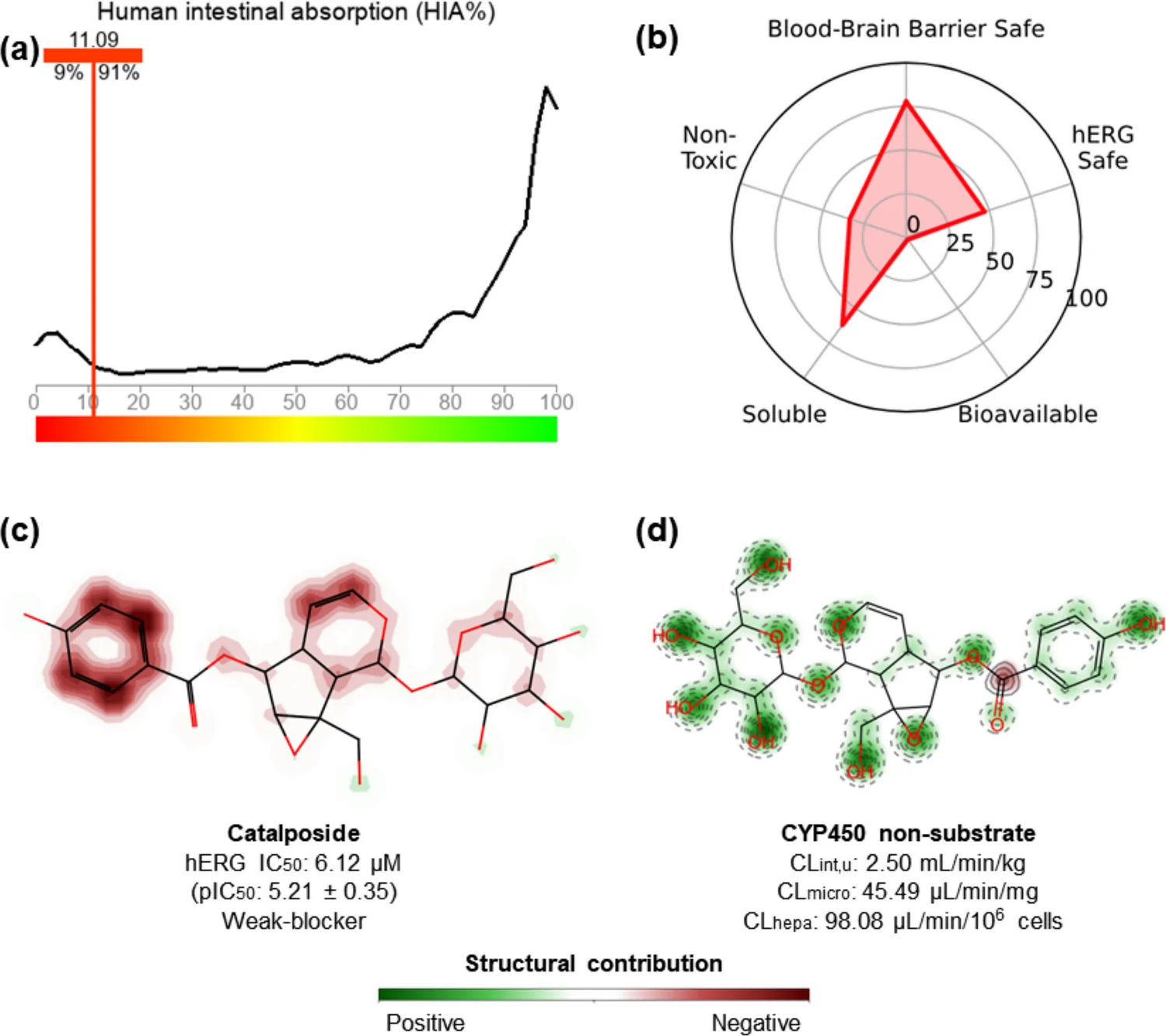
Safety and metabolism profile of catalposide. (**a**) Prediction of human intestinal absorption (HIA), indicating a value of 11.09 percent. (**b**) Safety radar chart demonstrating the balance between permeability at the blood-brain barrier, hERG safety, bioavailability, solubility, and overall toxicity. (**c**) Structural contribution map for hERG channel inhibition (IC50: 6.12 µM), classifying the compound as a weak-blocker. Red spots indicate regions that contribute negatively to cardiac safety. (**d**) Biotransformation analysis indicating catalposide as a non-substrate of cytochrome CYP450. The intrinsic clearance (CLint,u), microsomal clearance (CLmicro), and hepatic clearance (CLhepa) values are provided, with the color map indicating the positive (green) or negative (red) structural contribution to metabolic stability

With the increase in Fsp^3^ from 0.59 to 0.64, Catalposide (Prototype 1) can be modified by Analog 2 through the alkylation of an OH group in the glycoside substructure, which is less sterically hindered compared to the others in the system, forming a more lipophilic ether derivative due to the increase in van der Waals surface area (Fig. 7). The increase in lipophilicity caused the calculated logP to shift from 0.69 to 1.02, resulting in a more permeable analog in biological membranes, with Papp Caco-2 increasing from 5.49 × 10⁻⁷ cm/s to 8.71 × 10⁻⁷ cm/s, indicating that the compound may have an intestinal absorption of less than 70%, according to the biopharmaceutical classification system [56]. Corroborating this, an HIA% of approximately 11.09% is predicted (Fig. 8a), showing a similarity in data with only 9.0% of compounds that have scaffolds that reduce oral drug absorption, especially those associated with glycoside derivatives [48, 57].

For more lipophilic compounds, it is expected that they will distribute better into biological tissues than into blood plasma [58], corroborating the trend observed by the structural modification of Catalposide (Fig. 7). The generic structure of the compound tends to be more confined to blood plasma, with an estimated VDss of 0.51 L/kg, where the addition of the alkyl group to the OH with the least steric hindrance increased this value to VDss = 0.62 L/kg. This value suggests that, although systemic distribution is predominant, there was an optimization in distribution to the organic phase.

In general, the structure of catalposide may be less lipophilic than ideally expected for drugs with high cell permeability, potentially resulting in a low volume of distribution, and exhibiting pharmacokinetic attributes susceptible to multiparametric optimization (Fig. 7).

#### hERG safety and metabolic stability

The AI-based ADMET test showed a pharmacokinetic profile based on low oral bioavailability and a low probability of exhibiting a satisfactory safety profile, in addition to a 50% reduction in hERG safety (Fig. 8b). Using a structural similarity model with reactive molecular fragments, it was observed that the Csp2-H-rich aromatic portion negatively contributes to a region capable of binding to the hydrophobic framework of hERG channels (red color spectra), while the Csp3-H portion, resulting from an Fsp3 of approximately 0.59, presents a lower risk of hERG inhibition, resulting in a predicted IC50 of 6.12 µM, classifying the compound Catalposide as a moderate hERG blocker [55], while the regression model estimated a pIC50 on the order of 5.21 ± 0.35 µM (Fig. 8c). This risk is reduced by the presence of positive signals (green color) associated with the less sterically hindered OH groups (Fig. 8c).

On the other hand, the compound showed only one negative sign associated with the central carbon of the ester group (RO–C = O), as a group susceptible to hydrolysis by first-pass metabolic enzymes (in the human liver). However, the compound is mostly resistant to phase I metabolism dependent on CYP450 isoforms (Fig. 8d). The calculated CLint,u descriptor of 2.50 mL/min/kg, predicted by ADMET, suggests that the compound has an intrinsic clearance with slow release in the blood plasma, ensuring a longer half-life. According to multiparametric classification systems from Pfizer, Inc., [59, 60], Microsomal clearance indices (CLMicro) starting at 50 µL/min/mg can already cause a reduction in the oral bioavailability of drug candidates, which occurs for Catalposide due to the predicted CLMicro of 45.49 µL/min/mg, indicating rapid clearance from the hepatic microsome system, supported by high passive efflux from hepatocytes, with a predicted CLHepa of 98.08 µL/min/106 cells.

## Conclusion

This theoretical study indicates that catalposide, an iridoid glycoside derived from *Veronica anagallis-aquatica L*., stands out as a promising drug prototype for treating erectile dysfunction. Through a robust approach combining molecular docking and 500 ns molecular dynamics, the compound exhibited superior predicted binding affinity (−8.6 kcal/mol) and greater structural stability regarding the therapeutic target PDE-5 compared to the reference drug Vardenafil. Crucially, thermodynamic and trajectory analyses strongly suggest high selectivity for PDE-5 over PDE-6 (an off-target). While Vardenafil bound strongly to and stabilized the PDE-6 structure in silico, catalposide did not restrict PDE-6's structural flexibilitym imicking the unstable nature of the free apoenzyme and showed an almost total absence of a stable intermolecular hydrogen-bonding network. These observations were quantitatively corroborated by Interaction Potential Energy (IPE) calculations, which predicted a highly spontaneous and favorable binding free energy for the catalposide-PDE-5 complex (ΔG_bind_ = −27.05 kJ/mol), in direct contrast to its theoretically unfavorable interaction with PDE-6 (ΔG_bind_ =  + 4.67 kJ/mol). This weak in silico affinity for PDE-6 implies a reduced risk of cross-reactivity, potentially avoiding visual side effects such as cyanopsia. Despite its predicted pharmacodynamic efficacy and selectivity profile, ADMET analysis revealed limitations regarding oral bioavailability due to low intestinal permeability (11.09%). However, predictive analyses indicated that increasing the sp3 fraction through alkylation is a viable theoretical strategy to improve the compound's lipophilicity and absorption. In summary, these computational results highlight catalposide as a highly promising, potentially selective, and structurally optimizable lead compound, justifying future in vitro and in vivo experimental validation for the development of next-generation therapies for erectile dysfunction.

## Data Availability

All the data generated or analyzed during this study are included in this published article.
